# Home Use of a Pyrethroid-Containing Pesticide and Facial Paresthesia in a Toddler: A Case Report

**DOI:** 10.3390/ijerph13080829

**Published:** 2016-08-17

**Authors:** Alexandra Perkins, Frederick Walters, Jennifer Sievert, Blaine Rhodes, Barbara Morrissey, Catherine J. Karr

**Affiliations:** 1 Department of Environmental and Occupational Health Sciences, University of Washington, Seattle, WA 98105, USA; aperk11@uw.edu; 2 Northwest Pediatric Environmental Health Specialty Unit, Seattle, WA 98105, USA; 3 Bainbridge Pediatrics, Bainbridge Island, WA 98110, USA; fwalters@bainbridgepediatrics.com; 4 Washington State Department of Health, P.O. Box 47825, Olympia, WA 98504, USA; Jennifer.Sievert@doh.wa.gov (J.S.); Blaine.Rhodes@doh.wa.gov (B.R.); Barbara.Morrissey@doh.wa.gov (B.M.); 5 Department of Pediatrics, University of Washington, Seattle, WA 98105, USA

**Keywords:** pesticide, insecticide, pyrethroid, paresthesia, pediatric, child, biomonitoring

## Abstract

Paresthesias have previously been reported among adults in occupational and non-occupational settings after dermal contact with pyrethroid insecticides. In this report, we describe a preverbal 13-month-old who presented to his primary care pediatrician with approximately 1 week of odd facial movements consistent with facial paresthesias. The symptoms coincided with a period of repeat indoor spraying at his home with a commercially available insecticide containing two active ingredients in the pyrethroid class. Consultation by the Northwest Pediatric Environmental Health Specialty Unit and follow-up by the Washington State Department of Health included urinary pyrethroid metabolite measurements during and after the symptomatic period, counseling on home clean up and use of safer pest control methods. The child’s symptoms resolved soon after home cleanup. A diagnosis of pesticide-related illness due to pyrethroid exposure was made based on the opportunity for significant exposure (multiple applications in areas where the child spent time), supportive biomonitoring data, and the consistency and temporality of symptom findings (paresthesias). This case underscores the vulnerability of children to uptake pesticides, the role of the primary care provider in ascertaining an exposure history to recognize symptomatic illness, and the need for collaborative medical and public health efforts to reduce significant exposures in children.

## 1. Introduction

Low dose chronic pesticide exposures are common in the United States and around the world given widespread use in homes, gardens, and agricultural settings [1]. A population-based survey of households with young children found that over 80% reported applying some type of insecticide in the previous year [2]. Children have been identified as particularly vulnerable to uptake of pesticides from their environment due to frequent hand-to-mouth behavior, ingestion of soil and dust, mouthing of nonfood items, increased contact with soil, floors and carpets where spray residues settle, and higher concentrations of pesticide residues close to the floor in their breathing zone [3,4,5]. In the U.S., residential applications have been identified as the most important contributor to children’s exposure to pyrethroid insecticides [6].

We describe a case of pyrethroid insecticide toxicity in a toddler resulting from use of a common household insecticide product. Symptomatic pediatric pesticide poisonings are relatively rarely reported, especially in countries such as the U.S., where regulatory protections have reduced risk. However, it is likely some pesticide-related toxicity in children goes unrecognized due to the non-specific presentation of these illnesses.

## 2. Case History

A 13-month-old boy with normal development and no prior significant medical problems presented to his primary care pediatrician with a one-week history of persistent odd facial movements. His parents observed no other unusual signs or symptoms and reported he was otherwise behaving normally. The pediatrician observed the symptoms as somewhat tic-like. History taking revealed the patient’s family had been coincidentally treating an ant problem in their house (previous two weeks) using products they purchased and applied themselves as instructed on the label. They also reported hiring a licensed pest management professional (PMP) to treat their home (indoors and outdoors) during the same period. The child was not taking any medications and no unintentional exposure sources to medications or other toxic substances were identified. All other household members who included his parents and a 32-month old sibling were in good health without symptoms or health complaints. The pediatrician requested the label for the home use products. Given the rarity of tic disorders in the toddler period and the temporal relationship of the symptoms to pesticide use in the home, the physician consulted the Northwest Pediatric Environmental Health Specialty Unit (NW PEHSU) at the University of Washington (Catherine Karr) and a child neurology specialist. The pediatric neurologist found no abnormal findings beyond the facial movements and electroencephalogram (EEG) testing was normal. 

The NW PEHSU informed the pediatrician that the pesticide active ingredients identified on the label had known neurotoxicity and further investigation was merited. The family was advised that use of the product should be discontinued immediately. 

Suspected pesticide-related illness is a reportable condition in Washington State and NW PEHSU alerted the Washington State Department of Health (WDOH). The WDOH Pesticide Illness Monitoring and Prevention staff and NW PEHSU worked together to assess the exposure history by time, location, and active ingredient (see Table 1). The child’s parents were interviewed further, application records were obtained from the PMP, and medical records from the pediatrician were reviewed. The family was counseled to clean treated areas with soap and water, and steam clean carpeting to remove residues in the home based on the pesticide manufacturer recommendation. Symptoms resolved spontaneously in the days following home cleaning.

The expanded exposure history discerned multiple pesticide types and applications in the home (Table 1). Approximately one week before the symptoms developed, the family purchased and applied a product containing active ingredient d-limonene (5%) but found it ineffective. One week later, the licensed PMP applied fipronil (0.06%) to the foundation and applied chlorfenapyr (0.5%) and imidacloprid (0.05%) inside as crevice treatment in the kitchen and master bath. An ant bait gel containing sodium tetraborate decahydrate (5.0%) was applied to the window sills of the master bath. Two days after the PMP application, the parent purchased an indoor/outdoor ready-to-use insecticide containing pyrethroids bifenthrin (0.05%) and zeta-cypermethrin (0.0125%). This spray product was applied at night to the kitchen, living room, master bath and along baseboards in the child’s carpeted play room. 

Onset of the toddler’s facial movements was noted the day after first use of this home spray pyrethroid insecticide. The product was sprayed several more times over the following week coincident with the persistence of the child’s facial symptoms. The PMP returned the next week and applied a second pyrethroid product to the foundation of the home (bifenthrin 7.9%). Indoors, the PMP applied an insecticide containing pyrethrins, piperonyl butoxide, and amorphous silica, as well as the same gel bait applied the week before along the window sills of the master bath.

NW PEHSU suspected that the symptoms could represent facial paresthesias caused by dermal contact with the pyrethroid home spray applied to baseboards in the carpeted playroom and other areas of the house. Such manifestations had been reported in several case reports of adults following both occupational and non-occupational exposures to pyrethroid-containing insecticides and their volatilized form [7,8,9]. NW PEHSU requested that the WDOH biomonitoring program provide analysis of the patient’s urine for pyrethroid metabolites. Pyrethroids are metabolized and excreted rapidly in humans and urinary metabolites provide a measure of recent exposure. The WDOH program had recently conducted pyrethroid metabolite biomonitoring in a general statewide population sample including a sample of children aged 6–11 years. WDOH agreed to test the patient’s urine. The analytical method used was based on the Centers for Disease Control and Prevention (CDC) method for pyrethroids in urine (CDC 2013). Briefly, the analytical method enzymatically decoupled the pyrethroid-sugar complexes in the patient’s urine and separated the pyrethroid metabolites from the fluid using solid phase extraction. The purified solution of mixed metabolites was injected into a High Pressure Liquid Chromatography (HPLC) and the individual metabolites separated by the HPLC column were identified, quantified, and confirmed by tandem mass spectrometry. The available test panel included 3-phenoxybenzoic acid (3-PBA) and *trans*-(2,2-dichlorovinyl)-2,2-dimethylcyclopropane-1-carboxylic acid (*trans*-DCCA), metabolites common to *zeta*-cypermethrin and several other pyrethroid insecticides. There is no available metabolite testing for bifenthrin in the U.S. The available test panel did not include any of the other non-pyrethroid pesticides applied in this home. 

A spot urine sample collected from the patient on day six of the symptomatic period showed urinary metabolite concentrations of 2.22 µg/g creatinine (Cr) for 3-PBA and 3.82 µg/g Cr for *trans*-DCCA. These levels were in the range of the 90th and 95th percentile observed for a representative sample of young school age children during a relatively recent Washington State survey (age 6–11 years), respectively (Table 2). 

Exposures to the other pesticides used in the home have not been associated with paresthesias and applications were done in a manner that would present less opportunity for the child’s exposure (e.g., crack and crevice treatment, gels, outdoor foundation treatments) compared to repeated spray application of the home-use pyrethroid product in areas where the child spent a significant amount of time (sprays along baseboards in the carpeted playroom).

Follow up urine testing seven weeks later, in the non-symptomatic period, showed a significant drop in 3-PBA and *trans*-DCCA metabolites to below the 50th percentile range of the reference sample (see Table 2).

A diagnosis of pesticide-related illness due to pyrethroid exposure was made based on the opportunity for significant exposure (multiple applications in areas where the child spent time), supportive biomonitoring data, and the consistency and temporality of symptom findings (paresthesias).

## 3. Discussion

To our knowledge, this is the first child case report of pyrethroid pesticide toxicity manifesting in facial paresthesias. It illustrates several key points including the particular vulnerability of young children to commonly used pest control products including toxicity under use conditions described on the label. Furthermore, the important role of the health care provider in recognizing potential toxicity and the collaborative public health role in surveillance and prevention are demonstrated. 

Pyrethroids are a class of neurotoxic insecticides used widely for agricultural and residential pest control. Toxicity testing identifies multiple nervous system targets in mammalian systems, including voltage-gated sodium and chloride channels, and gamma-aminobutyric acid (GABA), nicotinic acetylcholine, and peripheral benzodiazepine receptors [8]. The pyrethroids used in this child’s home, cypermethrin and bifenthrin, have generally low systemic toxicity via dermal contact and inhalation but moderate to high acute toxicity if ingested. Absorption across intact skin is low [5,11,12]. Notably, topical contact with pyrethroids is associated with paresthesias, which are believed to result from local action on sensory neurons in the skin [13]. Paresthesias, which manifest as stinging, itching and numbness commonly in the face, have been observed in the absence of other pyrethroid toxicity symptoms in occupational case reports [8,14,15,16]. This preverbal child’s odd facial movements were suspected to represent a response to these well-described paresthesias. In general, paresthesias dissipate within 24 h of removal from the exposure source and in this case, symptoms resolved in the days following home cleaning to remove remaining residues [8].

Young children are at higher risk of exposure than adults following use of indoor pesticide sprays. After spray application, pesticide residues settle on floors and surfaces, which contributes to a higher risk of dermal contact for children who crawl and play on the floor [3]. Younger children exhibit the highest extent of hand to mouth and mouth to object behavior, which can increase exposure to residential pesticide residues [4,17]. Children take in more air on a per kilogram basis than adults, so when air contains volatilized pesticides or dust containing pesticides, they receive a higher dose. Spraying of baseboards in the playroom provided a significant source of exposure for this toddler.

Partly due to their more favorable (less acute) toxicity profiles, pyrethroids have replaced organophosphorus insecticides in residential pest control products [18]. They are among the most commonly used and stored class of pesticide in U.S. homes and are among the most commonly identified pesticide residues on household surfaces [19]. They also represent the class associated most frequently with pesticide exposures in children reported to U.S. network of Poison Control Centers [20]. 

In the case presented, multiple pesticides were applied in and around the child’s home on at least 6 different days in a two-week period. This case illustrates the need for raising awareness of the health risks associated with pesticides, especially to children. Greater education is needed for consumers seeking do-it-yourself pest control. For example, integrated pest management (IPM) methods which prioritize no or low toxicity approaches are recognized for their effectiveness as well as safety [21,22]. 

In this case, state-based public health resources for biomonitoring and investigation were helpful but unfortunately are not available to clinicians in every setting. In the U.S., suspected pesticide-related exposure is a reportable condition in all but 13 states (reporting is optional in 6 states) [23]. Such programs provide useful public health tracking as well as individual case support. A more comprehensive national or global pesticide-related illness surveillance system would greatly enhance our understanding of the magnitude of pesticide-related illnesses in children. 

The ability of healthcare providers to take an environmental history, read pesticide labels, to identify symptoms of poisoning, and to provide anticipatory guidance is a critical part of efforts to prevent unnecessary and potentially harmful exposures. Unfortunately, data suggests that most pediatricians in the U.S. are poorly prepared for this. Only 12% of chief residents in pediatric residencies surveyed in 2003 reported pesticide content was part of their curriculum [24]. A 2006 survey of healthcare providers in a highly productive agricultural area with high pesticide use revealed that only 30% had training on pesticides and children’s health [25]. This illustrates the need for knowledge of pesticides and their health effects in medical education as well as accessible specialty consultation resources [26]. In North America, the network of academically-based Pediatric Environmental Health Specialty Units (PEHSUs) are available for consultations on non-emergent management and questions related to low dose, chronic environmental exposures while the Poison Control Centers remain the primary source of guidance on acute poisoning management in most settings [27].

Pyrethroid biomonitoring was available in this case but its usefulness for case diagnosis is subject to a few limitations. These urinary metabolites are an indicator of exposure only. There is no established threshold of exposure associated with symptom onset. Elevated biomarkers may be associated with diverse sources of pyrethroids including: background dietary exposure, lice and scabies treatments, public mosquito spraying programs, or mosquito resistant clothing. In the case presented, dietary exposure could not be ruled out. Finally, spot urine measurement of rapidly excreted metabolites can be highly variable throughout a day. While the elevated 3-PBA metabolite in this case report is consistent with increased exposure, it cannot alone confirm that the child’s symptoms were caused by the pyrethroid, nor that the pesticides sprayed in the home were the source of elevated pyrethroids in the child’s urine. In this case, the urine tests were supportive but not confirmative of the diagnosis. Diagnosis relied on patient history, presence of a hallmark sign, supportive urine testing, and the ruling out of other etiologies.

## 4. Conclusions

We report a clinically significant exposure to home-use pyrethroid insecticide in a toddler. The case illustrates the unique vulnerability of children to routine pesticide exposure and the frontline role of the pediatric health professional in recognizing toxicity through taking an environmental history. Once recognized, collaborative support of environmental medical and public health specialists can support clinicians in deciphering timely and appropriate diagnosis and thwarting ongoing exposure and potentially more significant health consequences (secondary prevention). This case also illustrates the ongoing need for programs and policies to reduce pesticide applications in children’s environments (primary prevention). 

## Figures and Tables

**Table 1:** Pesticide active ingredient applications and symptom timeline.

	10/16/13	10/22/13	10/24–10/31/13	10/30/13	11/1/13
	Asymptomatic		Odd Facial Movements Develop and Persist, Resolve in First Week of November		
**Application by parent**	Indoor: d-limonene (5%)		Indoor: bifenthrin (0.05%) and zeta-cypermethrin (0.0125%) applied to play room, kitchen, living room, and master bath		Cessation of pesticide use
**Application by hired professional applicator**		Outdoor: fipronil (0.06%) applied to foundation; Indoor: chlorfenapyr (0.5%) and imidacloprid (0.05%) as crevice treatment in kitchen and master bathroom; sodium tetraborate decahydrate gel applied to window sill of master bath	Outdoor: bifenthrin (7.9%) applied to foundation; Indoor: pyrethrins, piperonyl butoxide, and amorphous silica as crevice treatment in master bathroom; sodium tetraborate decahydrate gel applied to window sill in master bath		

**Table 2:** Pyrethroid urinary metabolite concentrations (µg/g Creatinine).

	11/01/13 (Symptomatic)	12/20/13 (Symptoms Resolved)	WA Children, 2010–2011 ^3^ 6–11 Years 50th Percentile (95% CI)	WA Children, 2010–2011 ^3^ 6–11 Years 95th Percentile (95% CI)
**3-PBA ^1^**	2.22	0.329	0.53 (0.41–0.69)	7.47 (2.86–15.4)
***trans*-DCCA ^2^**	3.82	0.453	(<LOD–0.421)	2.61 (1.4–15.8)

^1^ 3-PBA (3-phenoxybenzoic acid) is a general metabolite of pyrethroid pesticides; ^2^ *trans*-DCCA (*trans*-(2,2-dichlorovinyl)-2,2-dimethylcyclopropane-1-carboxylic acid) is a metabolite of permethrin, cypermethrin, and cyfluthrin; ^3^ Washington (WA) environmental biomonitoring survey, May 2010–June 2011 [10]. Washington State Department of Health; CI: confidence interval; LOD: limit of detection.
